# Evaluating Provitamin A Carotenoids and Polar Metabolite Compositions during the Ripening Stages of the Agung Semeru Banana (*Musa paradisiaca* L. AAB)

**DOI:** 10.1155/2020/8503923

**Published:** 2020-05-12

**Authors:** Rosita D. Chandra, Chandra A. Siswanti, Monika N. U. Prihastyanti, Heriyanto, Leenawaty Limantara, Tatas H. P. Brotosudarmo

**Affiliations:** ^1^Ma Chung Research Center for Photosynthetic Pigments (MRCPP) and Department of Chemistry, Universitas Ma Chung, Villa Puncak Tidar N01, Malang 65151, Indonesia; ^2^Faculty of Biochemistry, Biophysics and Biotechnology, Jagiellonian University, Ul. Gronostajowa 7, 30-387 Krakow, Poland; ^3^Center for Urban Studies, Universitas Pembangunan Jaya, Jl. Cendrawasih Raya B7/P, South Tangerang, 15413 Banten, Indonesia

## Abstract

Banana cultivars that are rich in provitamin A carotenoids and other nutrients may offer a potential food source to help alleviate vitamin A deficiencies, particularly in developing countries. The local plantain type banana, Agung Semeru (*Musa paradisiaca* L.), was investigated, in order to analyse the changes in the compositions of the provitamin A carotenoids and metabolite compounds, including the amino acids, organic acids, and sugars, during the ripening stage as this banana is widely processed for food products in either the unripe, ripe, or overripe stages. The bananas that had reached the desired ripening stages were subjected to high-performance liquid chromatography (HPLC) analysis, and the results indicated that the total provitamin A carotenoid concentrations ranged between 4748.83 *μ*g/100 g dry weight (dw) and 7330.40 *μ*g/100 g dw, with the highest level of vitamin A activity at 457.33 ± 5.18 *μ*g retinol activity equivalents (RAE)/100 g dw. Compared to the Cavendish variety, which is consumed worldwide, the Agung Semeru banana had vitamin A activity that was 40 to 90 times higher, dependent on the stage of ripening. The breakdown of the starch during the ripening stages resulted in an increase of its sugar compounds, such as sucrose, fructose, and glucose, as well as its dominant organic acids, such as malic acid, oxalic acid, and citric acid, which were observed using gas chromatography-mass spectrometry (GC-MS) during the ripening stages. The findings of this study show that the Agung Semeru banana is a promising fruit that could be widely produced as a nutritional and energy food resource, due to its high levels of vitamin A activity and sugars.

## 1. Introduction

Nutritional deficiencies and malnutrition are still global problems, especially for the poor and the majority of developing countries, including Indonesia, leading to health problems such as skin diseases, defective bone growth, dementia, and increased mortality [1–4]. In Indonesia, vitamin A deficiency (VAD) is a nutritional deficiency that is considered mild and not a public health problem, as it impacts less than 10% of the population [5]. However, this must be interpreted with caution as the prevalence of VAD may differ from year to year [5]. In 2001, as an example year, 18% of mothers and 54% of infants were found to be VAD in Indonesia [3]. According to Atmarita [4], there are two categories of nutritional problems: (1) insufficient food intake and (2) excessive or unbalanced food intake. In Indonesia, the first category is the main reason for nutritional deficiencies, including VAD. Nevertheless, positive health behaviours and better eating habits could play an important role in improving the nutritional status. Consuming fruits or vegetables rich in provitamin A carotenoids is one alternative that can be employed to reduce the number of vitamin A deficiencies in Indonesia.

Bananas are a tropical fruit that grows abundantly in all regions of Indonesia and can thus be easily be obtained. Besides containing nutritional compounds including carbohydrates, proteins, sugars, and minerals, bananas also provide provitamin A carotenoids such as *α*-carotene and *β*-carotene that can be converted into vitamin A in the human body [6]. The vitamin A activity of carotenoids is indicated by retinol activity equivalents (RAE), and as described by the Institute of Medicine (U.S.), the conversion factor for *β*-carotene is 12, i.e., 1 *μ*g RAE is equal to 12 *μ*g *β*-carotene or 24 *μ*g of other provitamin A carotenoids, such as *α*-carotene and *β*-cryptoxanthin [7]. Therefore, bananas are considered a fruit that when regularly consumed could help to combat VAD, particularly in Indonesia.

Banana cultivars that are rich in provitamin A carotenoids may be a food source that could help alleviate VAD [8]. The most marketed banana cultivars, Cavendish type, contain low levels of provitamin A carotenoids, ranging from 21 to 290 *μ*g *β*-carotene/100 g dry weight (dw) [8–11]. An attempt to increase the level of provitamin A to 20 *μ*g/g of *β*-carotene has been achieved in transgenic Cavendish bananas [12]. In Southeast Asia, there are 149 local cultivars of edible bananas [13], and some have an average concentration of *β*-carotene that ranges from 230 to 1370 *μ*g/100 g dw [8, 9]. One variety of banana that is typical to Indonesia and of interest for further investigations is the Agung Semeru (*Musa paradisiaca* L. AAB plantain); as to the best of our knowledge, there have been no previous studies conducted to examine the content of the provitamin A carotenoids in this banana. Agung Semeru grows well at 450-650 m above sea level. The characteristic of this plantain include the colour of its pseudostem (light red), the number of suckers per cluster (only 1-2 suckers per cluster), the size of the fingers (33-36 cm long and 19 cm around), the number of hands per bunch (only 1-2 hands per bunch), and a weight of ~10-20 kg/bunch [14, 15]. It has been cultivated only in East Java, Indonesia, especially in the Lumajang regency, on more than 553 hectares of land, with an average production of 28 000 tons per year. The fruits have a long storage period of 3-4 weeks after harvesting, as the flesh can still be consumed although the peels do turn black [16, 17].

The Agung Semeru bananas are usually harvested at the mature green stage, as it is widely processed for food products in either the unripe, ripe, or overripe stages. Therefore, it is necessary to investigate the compositions of the provitamin A carotenoids during the different ripening stages. The peel colours are often used as the major indicator to determine fruit ripeness [18]. Several studies have focused on the carotenoid accumulations and chlorophyll degradation mechanisms in the peel of banana fruits during postharvest ripening [19, 20]. However, the natural ripening is a combination of physiological, biochemical, and molecular processes that lead to changes in the colour, sugar content, acidity, flavour, aroma, and carotenoid contents and compositions [21–26], impacting the quality of the banana. Thus, besides the importance to determine the amount and availability of the provitamin A carotenoids, other metabolite compounds from the early to the final stage of ripening should be evaluated to determine the quality of the banana and further improve human nutritional status. Hence, the aim of this study was to analyse the changes in the compositions of the provitamin A carotenoids and metabolite compounds, including the amino acids, organic acids, and sugars during the ripening stages of the Agung Semeru (*Musa paradisiaca* L.) plantain.

## 2. Materials and Methods

### 2.1. Plant Material and Preparation

**Figure 1:**
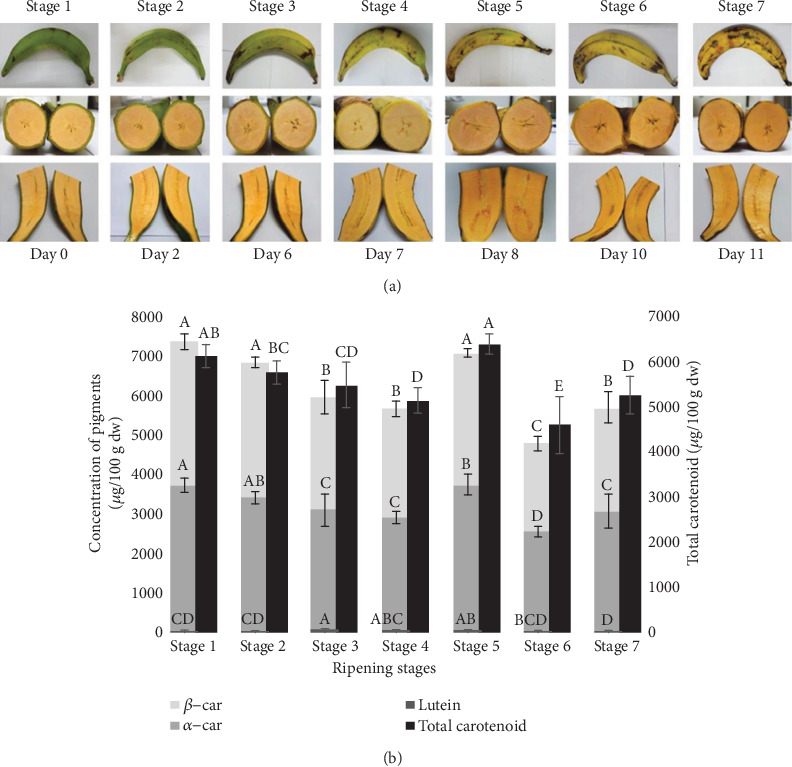
Changes in the carotenoids of Agung Semeru plantains during development. (a) Changes in the fruit colour during postharvest ripening. (b) Changes in the carotenoid content and composition of the fruit flesh during postharvest ripening, obtained from analysis using HPLC (*β*-car, *α*-car, and lutein) and spectrophotometry UV-Vis (total carotenoid). Different lowercase letters indicate significant differences among the ripening stages (*p* < 0.05).

Agung Semeru (*Musa paradisiaca* L. ABB group) plantain bunches from the Senduro banana plantation, Lumajang regency, East Java, were collected from a single batch harvest. Each bunch of the Agung Semeru plantains contained 10-14 fingers and did not have a hand. The samples were harvested during the rainy season and were selected when the fruits of the identified bunches were mature (deep green, full, and rounded) and standardized according to the size of the fruit, absence of physiological defects, and visual infections. The fruit samples were allowed to ripen naturally in a well-aerated and well-shaded room at ambient temperatures (25 ± 2°C). The ripening stages of the bananas were observed on the basis of the peel colour (Figure 1(a)): 1 = green; 2 = green with a trace of yellow; 3 = more green than yellow; 4 = more yellow than green; 5 = only green tips remaining; 6 = all yellow; and 7 = yellow flecked with brown [27]. Prior to the analysis, one finger from three different bunches that had reached the desired ripening stage was randomly detached for analysis in triplicate, by cutting with a knife at the base part of the fruit, without damaging the other fingers; then, they were cleaned and cut into three parts. The rest of the fingers were left attached to the bunch to ripen naturally in the same room conditions.

### 2.2. Carotenoid Extraction and HPLC Analysis

All extractions were carried out in triplicate according to previously reported protocols [28] with modifications, specifically for the analysis of the banana tissues. Fresh banana flesh aliquots (0.2 g) were homogenized for 5 min at maximum speeds on a vortex (IKA, Staufen, Germany) in 1.0 mL ice-cooled extraction solvent. The extraction solvent consisted of EtOH and *n-*hexane (4 : 3, *v*/*v*). The extract was kept cool on ice, and the extraction was carried out in a dark room. Following centrifugation at 5000 rpm for 1 min at 5°C, the supernatant was evaporated using a rotary evaporator (Heidolph, Schwabach, Germany). The residues were then redissolved in 1 mL acetone and then filtered using a filter membrane (PTFE, 0.2 *μ*m) prior to HPLC analysis.

Twenty *μ*L of each sample was used for the determination of the carotenoid content, following the method of Kurniawan et al. [29], using separation with a Shimadzu high-performance liquid chromatograph (HPLC) on a YMC carotenoid C-30 reversed-phase (Wilmington, MA, USA) column (150 × 4.6 mm I.D.), equipped with a guard column and photodiode array detector, using the elution gradient program of H_2_O : methanol : methyl tert-butyl ether (MTBE) (4 : 81 : 15, *v*/*v*/*v*) at 0 min and H_2_O : methanol : MTBE (4 : 6 : 90, *v*/*v*/*v*) at 70 min, with a flow rate of 1 mL/min at 30°C. The standard curves of the pigments, lutein, *α*-carotene (*α*-car), and *β*-carotene (*β*-car), were obtained by using the standard pigment manufactured by NATChrom (Malang, Indonesia). The linear equations were used to determine the concentrations of the lutein (*y* = 305.8*x*–0.6335, *R*^2^ = 0.9999), *α*-car (*y* = 232.04*x* + 47.906, *R*^2^ = 0.995), and *β*-car (*y* = 206.57*x* + 74.953, *R*^2^ = 0.9982) (where *y* is the peak area and *x* is the concentration of the determined pigment) in *μ*g/mL, at their maximum absorption wavelengths (*λ*_max_) of 445, 445, and 450 nm, respectively. The rest of the sample was then measured using the pigment absorption spectrum with the spectrophotometer (UV-1700, Shimadzu) for determination of the total carotenoids.

### 2.3. Determination of Total Carotenoid and Vitamin A Activity

Total carotenoids were measured using the following equation provided by Gross [30]:

(1) $$\begin{array}{c} \frac{\mu \text{g carotenoid}}{\text{g}}=\frac{A\times V\times 10^{6}}{A_{1\text{ cm}}^{1\%}\times 100\times G}, \end{array}$$

where *A* is the maximum absorbance, *V* is the volume (mL), *G* is the weight of sample (g), and *A*_1cm_^1%^ = 2500 for a specific absorbance extinction of a mixture of carotenoids in acetone.

The values obtained from Equation (1) were on a fresh weight (fw) basis, and the moisture content was determined using a Shimadzu MOC63u moisture analyser. Both results had to be converted into dry weight (dw) as follows:

(2) $$\begin{array}{c} \text{Total carotenoid }\left(\text{dw}\right)=\text{Total carotenoid }\left(\text{fw}\right)\times \frac{100}{100-\text{moisture content }\left(\%\right)}. \end{array}$$

After the measurement of the total carotenoids, the calculation of the vitamin A activity of the carotenoids in each stage of the banana was carried out by determining the concentrations of the provitamin A carotenoids, *α*-car and *β*-car, by using each standard curve formula. After that, the conversion of the provitamin A carotenoids to the vitamin A activity in the retinol activity equivalent (RAE) was calculated by using the newly advised conversion factor, as described by the Institute of Medicine (U.S.), of a 12 : 1 conversion from the *β*-car and a 24 : 1 from the *α*-car equivalents to RAE [7].

### 2.4. Analysis of the Composition of Amino Acids, Organic Acids, and Sugars by Gas Chromatography Coupled with Mass Spectrometry

The following method was a modification from a previously published method [31]. Bananas were frozen overnight prior to the lyophilization process (Labconco Co., USA) for 24 h at -45°C under high vacuum conditions (0.04 mbar). The lyophilized fruit samples (50 mg) from the different ripening stages were ground into fine powder, followed by extraction with 700 *μ*L methanol, with the addition of the internal standard (IS), 150 *μ*L ribitol (0.2 mg/mL in water) [32]. After extraction for 30 min at 70°C, they were vigorously mixed with 500 *μ*L water and 300 *μ*L chloroform, in order to separate the polar and nonpolar metabolites. The mixture was then subjected under centrifugation at 10 000 rpm for 5 min. Five hundred *μ*L of the upper phase which consisted of water/methanol was taken and dried using nitrogen gas. The residue was derivatized with 50 *μ*L *N*-methylhydroxylamine hydrochloride (20 mg/mL in pyridine) at 50°C for 30 min, followed by treatment with 100 *μ*L *N*,*O*-bis(trimethylsilyl)trifluoroacetamide at 60°C for 30 min. The sample volume of 0.2 *μ*L was injected into the GC column in a splitless mode.

The analysis using the GC-MS was performed on a Shimadzu QP2010 Plus instrument, operating in EI mode at 70 eV. The column used was Rtx-5MS (30 m × 0.25 mm × 0.25 *μ*m). Helium (GC-MS grade) was used as the carrier gas, flowing at a rate of 3 mL/min. The temperatures of the column oven and the injection were set at 40°C and 280°C, respectively. The temperature program for the analysis was performed as follows: 5 min at 40°C followed by a ramp of 20°C/min up to 180°C, and this was held for 3 min. The second ramp was 5°C/min up to 280°C and was held for 5 min. Meanwhile, the mass spectrum, which was recorded at 2 scans/s, was set in the scanning range, from 40 to 550 *m*/*z*. The temperatures of the interface and the ion sources were adjusted to 280°C and to 230°C, respectively.

### 2.5. Multivariate Data Analysis and Statistical Analysis

Means ± SE of each pigment compound from the ripening stages were statistically assessed from three replicates by mean comparison through a Tukey test using Minitab software version 17 (Minitab, State College, PA, USA). The mean differences within a 95% confidence interval (*p* < 0.05) were considered significant differences among the ripening stages.

Spectral data files of GC-MS were processed with the Wiley Registry library software (*Wiley Registry™ of Mass Spectral Data*, 9th edition, Ringoes, New Jersey) for metabolite identification. The detected metabolite peaks were identified by the software, and the extracted compounds were aligned according to the internal standard ribitol. Peaks with a similarity index of more than 80% were used for feature identification. After review, the relative concentrations based on the peak area of the compounds were normalized into a dry weight basis (dw), according to Equation (2). For further data analysis, using R software version 3.4.0, a principal component analysis (PCA) was used to visualize the data in accordance with the different ripening stage samples.

## 3. Results and Discussion

### 3.1. Carotenoid Analysis and Its Changes during Postharvest Ripening

The ripening changes in the Agung Semeru plantains were assessed in terms of the colour of the peel and flesh and the time that it took for each stage to be observed. Figure 1(a) shows the colour changes in the fruit flesh, which tended to become more orange with time, following the colour change in the peel. Stages 3 to 7 occurred between days 6 and 11, which was considered a rapid change. A previous study [15] determined that the storage duration of the Agung Semeru plantains was around 3 to 4 weeks postharvest, as the flesh was not too tender and still edible, although the peel colour turned dark brown and black.

The strong orange colour occurred in stage 5, and this stage contained the highest amounts of total carotenoids (Figure 1(b)). However, although the colour of the flesh at stages 6 and 7 looked like that of stage 5, the total carotenoid contents were considerably lower. Interestingly, stage 1 had the second highest total carotenoid content after stage 5, even though the colour of the flesh still looked pale.

Besides observing the colours of the plantain, the carotenoid pigments including *α*-car, *β*-car, and lutein, which were the main pigments in the banana, were determined and compared with the total carotenoid content. Previously, the major carotenoids identified in ripe bananas were lutein, *α*-car, and *β*-car, detected at 445, 451, and 453 nm [33]. Figure 1(b) represents the total concentration of the *α*-car, *β*-car, and lutein in each ripening stage, obtained from the calibration curve calculations, using high-performance liquid chromatography (HPLC).

**Figure 2:**
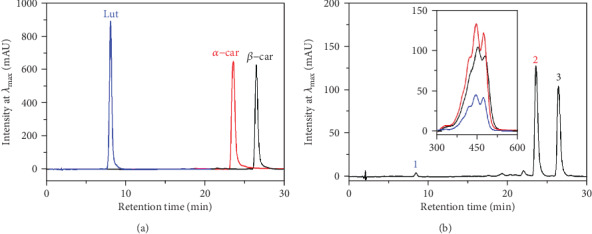
HPLC chromatogram profile of the standard lutein, i.e., *α*-car and *β*-car (a), and the extracts of the Agung Semeru plantain fruits at ripening stage 5 (b). The inset figure is the absorption spectra of the lutein (blue), *α*-car (red), and *β*-car (black) in the HPLC eluent.

For the identification of the pigments in the Agung Semeru plantains during the ripening stages, cochromatography between crude carotenoid extracts and carotenoid standards, which were lutein, *α*-car, and *β*-car, was applied to verify the identification results. It was in relation to its spectroscopic and chromatographic properties, such as the shape of the absorption spectrum, the position of the absorption maxima (*λ*_max_), and the retention time (*t*_R_), compared to the reference.  Figure 2 shows the chromatogram of stage 5 which was selected to represent all the ripening stages as it presented the highest amounts of total carotenoids, despite the changes in the concentrations of each pigment during the ripening stages. The HPLC separation profile contains three well-resolved compounds. The first compound, peak #1 eluted at a *t*_R_ of 8.3 min, corresponds to lutein. The second and third compounds, peaks #2 and #3 eluted at retention times of 23.5  and 26.4 min, correspond to *α*-car and *β*-car, respectively.

The content of the *α*-car, *β*-car, and lutein during the ripening stages (the data are clearly shown in Supplementary Table 1) were significantly different (*p* < 0.05), in the range of 2509.88 ± 5.49 *μ*g/100 g dw to 3684.79 ± 4.51 *μ*g/100 g dw for *α*-car whereas *β*-car ranged from 2238.94 ± 7.79 *μ*g/100 g dw to 3645.61 ± 5.53 *μ*g/100 g dw, with the highest levels of *α*-car and *β*-car obtained in stage 1. The ratio of the *α*-/*β*-car presented in stages 1 to 7 was 1.011, 0.997, 1.059, 1.033, 1.103, 1.121, and 1.157, respectively, which shows that a high ratio of *β*-car was rare and was only found at stage 2, whereas the ratio of *α*-car constantly increased from stage 5, reaching its highest ratio at stage 7. Meanwhile, the lutein which had the lowest pigment content, was presented in the range of 39.11 ± 11.78 *μ*g/100 g dw to 93.49 ± 21.13 *μ*g/100 g dw, in which the highest amount was found in stage 3 and the lowest in stage 7.

The concentrations of provitamin A carotenoids (*α*-car and *β*-car) were converted into vitamin A activity, and the retinol activity equivalents (RAE) ranged between 291.16 ± 20.06 *μ*g RAE/100 g dw and 457.33 ± 23.71 *μ*g RAE/100 g dw, with the highest vitamin A activity at stage 1, but it was not significantly different from that at stage 5 (430.96 ± 18.84 *μ*g RAE/100 g dw) (shown in Supplementary Table 1). The recommended dietary intake (RDI) values for vitamin A are 700 *μ*g RAE for females and 900 *μ*g RAE for males [34]. This suggests that by consuming 150 to 300 g of Agung Semeru bananas, depending on the ripening stage, the daily requirement of vitamin A could be fulfilled. In other local varieties of banana (Cavendish, Candi, Berlin, Raja, and Mas) that we analysed (unpublished data), at ripening stage 7, the vitamin A activity was 5.35 ± 0.80 *μ*g RAE/100 g dw, 70.11 ± 20.19 *μ*g RAE/100 g dw, 93.66 ± 12.51 *μ*g RAE/100 g dw, 95.13 ± 7.77 *μ*g RAE/100 g dw, and 118.02 ± 46.84 *μ*g RAE/100 g dw, respectively. This shows that the amount of vitamin A activity in the Agung Semeru banana was 2 to 90 times higher than that in all of these aforementioned bananas. The results also show that the Cavendish variety, which is consumed worldwide, provided the lowest amount of vitamin A activity, requiring about 14 000 to 18 000 kg to fulfil the daily need of vitamin A. Hence, the Agung Semeru banana has promise as a fruit that may be able to fulfil our daily requirements of vitamin A.

The total concentrations of *α*-car, *β*-car, and lutein were compared to the total carotenoid measured using a spectrophotometer, which was also determined in this study, and there was a significant difference in the results (Figure 1(b)). At the seven ripening stages, the total carotenoids ranged from 4609.29 ± 253.78 *μ*g/100 g dw to 6405.03 ± 87.37 *μ*g/100 g dw, with the highest amount obtained in stage 5, but it was not significantly different from that of stage 1. The differences or gaps in the results found between the two methods were probably caused by the presence of the minor pigments, which were detected in all the ripening stages of the banana. Those minor pigments were classified into four groups, oxidized carotene group, *cis*-*α*-carotene group, *cis*-*β*-carotene group, and a not identified carotene group, which contributed around 4.24 to 9.56% to the total of the carotenoid pigments. In addition, the different results might be due to the more specific pigments targeted in the determination using HPLC, while in that using a spectrophotometer all the carotenoids in the plantain were calculated. Besides that, the use of the epsilon value (*A*_1cm_^1%^) in the total carotenoid calculation, which is not specific for one dilution solvent, may also influence the results.

### 3.2. Analysis of Polar Metabolites during Postharvest Ripening

Polar metabolites of the Agung Semeru plantain were subjected to GC-MS analysis, and ~100 peaks were resolved, but only 27 peaks were picked for compound identification. The chosen peaks were identified based on the similarity of the retention indexes with the mass spectral libraries of the WILEY09, with similarities ≥85% (Supplementary Table 2). Most of the compounds were classified in the primary and secondary metabolites including sugar, organic acids, and amino acids, with multiple peaks provided by oxalic acid, fructose, glucose, and maltose. Approximately 13 peaks were identified at stages 1 and 2, 17 at stages 3 and 4, and 16 at stages 5 to 7 (Supplementary Table 2).

A typical GC-MS chromatogram of the polar metabolite compounds of ripening stage 5 can be seen in Supplementary Figure 1, as a representative example for the Agung Semeru plantain. It was chosen as the highest levels of total carotenoids were found at this stage. The peaks shown in the chromatogram were identified in Supplementary Table 2. The classes of compounds emitted most by the plantain at ripening stage 5 were the sugars, as they completely dominated the spectra at this stage, detected at 17 min (peak #10), while all the organic acids were not found in all the ripening stages. Oxalic acid was found in stages 1 and 2, while methylmalonic acid was detected in stages 3 and 4. Moreover, the malic acid and citric acid were found at all ripening stages, whereas the phosphoric acid was not presented at stage 3.

**Figure 3:**
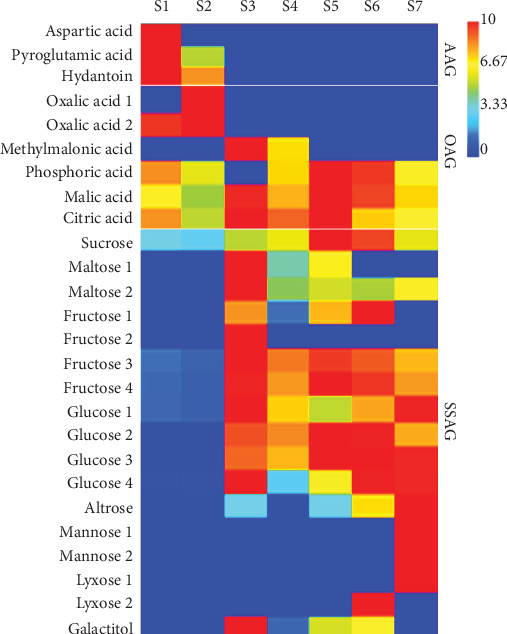
Polar metabolite levels, representatives of the major groups that changed during the development of the Agung Semeru plantain fruits. The amounts were based on the area of each peak which was normalized to the dry weight of each ripening stage (area/g). The ratio of the metabolite content at each ripening stage to the average metabolite content across the seven ripening stages is shown by the colour scale; the lowest ratio is in dark blue, and the highest ratio is in dark red. AAG = amino acid group; OAG = organic acid group; SSAG = sugar and sugar alcohol group.

To clearly highlight the differential distributions of the polar metabolites from the immature fruits (stage 1) to the overripe fruits (stage 7), a heat map is provided in Figure 3. Sucrose, maltose, fructose, and glucose were detected in this plantain as the major sugars. According to Arena et al. [35], the sucrose, glucose, and fructose have been found to be the most abundant carbohydrates, widely distributed in plants, with considerable variations in their ratios during the ripening stage. Maltose, fructose, and glucose began to accumulate at stage 3 and sucrose at stage 5. Sucrose was previously found to be the predominant sugar in the green stage of the banana [36], and this is in agreement with the findings presented here. Sucrose was found at the level of 32.21 × 10^6^ area/g dw at stage 1 and 29.31 × 10^6^ area/g dw at stage 2, continuing to increase by three times (92.10 × 10^6^ area/g dw) at stage 5 and gradually decreasing afterward. Fructose and glucose were also found to be dominant sugars in the earlier stages of ripening (stages 1 and 2), with the total fructose being 12.56 × 10^6^ area/g dw in stage 1 and 9.60 × 10^6^ area/g dw in stage 2. The total glucose in stage 1 was similar to that of fructose, while at stage 2 the amount of glucose was four times lower than that at stage 1. At stage 5, fructose and glucose were available in similar amounts, 107.99 × 10^6^ area/g dw and 114.41 × 10^6^ area/g dw, respectively. While continuing to ripen, the level of fructose decreased by half at stage 7, while the glucose kept rising to 140 × 10^6^ area/g dw. At stages 6 and 7, altrose, mannose, and lyxose began to accumulate, with the predominance of glucose.

Beside the presence of sugars, organic acids were also found as the dominant polar metabolite compounds in the Agung Semeru plantain. Organic acids are natural compounds commonly presented in banana flesh which play an important role in determining the quality of the banana [37]. Malic acid and citric acid have been found to be the most abundant organic acids in several fruits, including bananas [37, 38]. In this plantain, the concentrations of the dominant major acids, such as oxalic acid, malic acid, citric acid, phosphoric acid, and methylmalonic acid, changed markedly during the development and maturation. In the unripe stage (stages 1 and 2), the oxalic acid was the predominant acid, presented at the amount of 101.05 × 10^6^ area/g dw at stage 1 and 175.12 × 10^6^ area/g dw at stage 2, and it was not detected afterwards. According to Noonan and Savage [39], oxalic acid is present in several plant tissues and can impact human health, as the oxalates bind calcium and other minerals. In addition, excessive consumption of oxalic acids can cause stones to form in the urinary tract, as the acid is excreted in the urine. Meanwhile, methylmalonic acid was first found at stage 3, with far lower amounts compared to the oxalic acid, 857.11 × 10^3^ area/g dw, dramatically falling by half at stage 4 and not found afterwards. Malic acid, citric acid, and phosphoric acid were the organic acids that appeared in all ripening stages. As the banana ripens, the amounts of malic acid, citric acid, and phosphoric acid increased and reached their peaks at stage 5, with approximately 20 × 10^6^ area/g dw (for malic acid and citric acid) and 6.52 × 10^6^ area/g dw (for phosphoric acid), and they dropped afterwards. The results for oxalic acid, malic acid, and citric acid are in agreement with a previous study [40], which reported that oxalic acid was the predominant acid in the unripe stage of bananas and continuously declined as the banana ripened, while concentration of malic acid and citric acid increased and then started to decline when an overripe stage was reached. The decrease of the organic acids in the later stages might be due to the use of these compounds as a substrate for respiratory processes and the generation of ATP (adenosine triphosphate) during development and maturation [41].

In the early ripening stages, two compounds were clearly observed. Aspartic acid appeared in the first stage, while pyroglutamic acid was identified in stages 1 and 2. At stage 1, the level of aspartic acid was 2.46 × 10^6^ area/g dw, whereas the pyroglutamic acid was two times lower. At stage 2, the pyroglutamic acid was decreased by half, while the aspartic acid was not detected. These findings indicate that this plantain provides a limited composition of amino acids which can only be found at the unripe stage. Furthermore, hydantoin, which is a precursor of amino acids [42], was also found in the unripe stages, at around 40 × 10^3^ area/g dw in stages 1 and 2. This is interesting as no previous investigations have revealed that this compound is present in bananas or other fruits.

**Figure 4:**
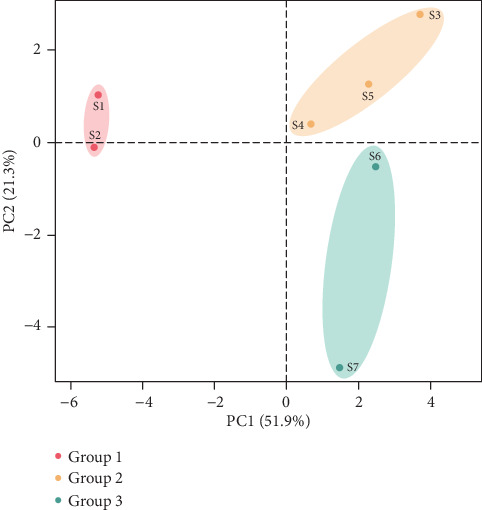
Principal component analysis (PCA) score plot of the identified polar metabolite compounds from seven ripening stages of the Agung Semeru plantains. Different coloured dots represent clustered stages in a group. Each stage is represented by averages of triplicates. S1: stage 1; S2: stage 2; S3: stage 3; S4: stage 4; S5: stage 5; S6: stage 6; S7: stage 7.

The cluster patterns of the polar metabolites were observed using PCA. The PCA as part of the multivariate analysis was conducted for the set of 27 metabolite compounds, in order to investigate the differences of the metabolites among the ripening stages and to determine the close clusters for each ripening stage.  Figure 4 shows the separation of seven ripening stages in PC1 and PC2, which accounted for 51.9% and 21.3% of the total variance, respectively. Stages 1 and 2 were tightly clustered together in the negative side of the PC1 as group 1 and were clearly separated from the other five ripening stages. This suggests closely related or identical metabolite compounds in stages 1 and 2, which might be contributed by the amino acid groups and oxalic acid. Stages 3 to 5 and stages 6 and 7 were grouped together on the positive side of the PC1, as groups 2 and 3, respectively. However, it can also be seen that the position of stage 6 was close to the cluster for group 2, which may be because the metabolite compounds of stage 6 were a transition between group 2 and stage 7. This might be contributed by the presence of sugars and organic acids (phosphoric acid, citric acid, and malic acid), which were also predominant until stage 6, while at stage 7 organic acids were reduced and there were new sugar groups detected, such as mannose and lyxose.

## 4. Conclusions

Evaluations of the provitamin A carotenoids and the polar metabolite compositions in the Agung Semeru banana (*Musa paradisiaca* L. AAB) found that there were high amounts of provitamin A carotenoids, due to the predominance of the *α*-car and *β*-car, with organic acids and sugars as the predominant polar metabolites. Stages 1 and 5 had the highest amounts of provitamin A carotenoids, which contributed to the levels of vitamin A activity. Compared to the Cavendish type, the Agung Semeru banana provided vitamin A activity that was 60 to 90 times higher and was able to fulfil a person's daily requirements, if 150 g was consumed when it was in stage 1 or 5 and 300 g in stage 6. Malic acid and citric acid were the predominant organic acids that were found in all ripening stages, while oxalic acid and methylmalonic acid were only present in the earlier stages. Lower levels of oxalic acid indicate a better quality of banana plantain. Moreover, sucrose, glucose, and fructose, collectively referred to as sugars, were found in all ripening stages. The sugars gradually decreased with time, except for glucose, which continued to increase and became the dominant sugar in stage 7, accompanied by the accumulations of new sugars, mannose and lyxose. This shows that the Agung Semeru banana is a promising fruit that could be widely produced as a nutritional and energy food resource, due to its high levels of vitamin A activity and sugars.

## Data Availability

All data generated or analyzed during this study are included in this published article and its supplementary material files.

## Supplementary Materials

Supplementary Materials Supplementary Table 1: carotenoid concentrations, total carotenoids, and vitamin A activity in the Agung Semeru plantain at all ripening stages, resolved using HPLC and spectrophotometry UV-Vis. Supplementary Table 2: polar metabolite compounds from the Agung Semeru plantain at all ripening stages, resolved by gas chromatography-mass spectrometry (GC-MS). Supplementary Figure 1: a typical separation profile of the targeted polar metabolite compounds from the Agung Semeru plantain in ripening stage 5, resolved by gas chromatography-mass spectrometry (GC-MS).
