# Supplementary material for: Evaluating Provitamin A Carotenoids and Polar Metabolite Compositions during the Ripening Stages of the Agung Semeru Banana (Musa paradisiaca L. AAB)
Source: Int J Food Sci. 2020 May 12;2020:8503923. doi: 10.1155/2020/8503923 (PMC7240789; doi:10.1155/2020/8503923)
Supplement: Supplementary Materials — Supplementary Table 1: carotenoid concentrations, total carotenoids, and vitamin A activity in the Agung Semeru plantain at all ripening stages, resolved using HPLC and spectrophotometry UV-Vis. Supplementary Table 2: polar metabolite compounds from the Agung Semeru plantain at all ripening stages, resolved by gas chromatography-mass spectrometry (GC-MS). Supplementary Figure 1: a typical separation profile of the targeted polar metabolite compounds from the Agung Semeru plantain in ripening stage 5, resolved by gas chromatography-mass spectrometry (GC-MS). [file 8503923.f1.zip › mat.8503923.v2.docx]

# Evaluating pro-vitamin A carotenoids and polar metabolite compositions during the ripening stages of Agung Semeru banana (*Musa paradisiaca L.* AAB)

Rosita D. Chandra^1^, Chandra A. Siswanti^1^, Monika N. U. Prihastyanti^1^, Heriyanto Heriyanto^1,2^, Leenawaty Limantara^3^, and Tatas H. P. Brotosudarmo^1^

^1^ Ma Chung Research Center for Photosynthetic Pigments (MRCPP) and Department of Chemistry, Universitas Ma Chung, Villa Puncak Tidar N01, Malang 65151, Indonesia.
^2^ Faculty of Biochemistry, Biophysics and Biotechnology, Jagiellonian University, ul. Gronostajowa 7, 30-387 Krakow, Poland.

^3^ Center for Urban Studies, Universitas Pembangunan Jaya, Jl. Cendrawasih Raya B7/P, South Tangerang 15413, Banten, Indonesia.

Correspondence should be addressed to Tatas H. P. Brotosudarmo; tatas.brotosudarmo@machung.ac.id

**Supplementary data**

Supplementary Table 1. Carotenoid concentrations, total carotenoids, and vitamin A activity in the Agung Semeru plantain at all ripening stages, resolved using HPLC and spectrophotometry UV-Vis.

| **Ripening stages** | **Pigment concentrations (µg/100 g dw)** | | | **Total carotenoids (µg/100 g dw)** | **Vitamin A activity (µg RAE/100 g dw)** |
| --- | --- | --- | --- | --- | --- |
|  | **Lutein** | ***α*-car** | ***β*-car** |  |  |
| Stage 1 | 48.38 ± 6.64^cd^ | 3684.79 ± 166.33^a^ | 3645.61 ± 201.50^a^ | 6128.73 ± 253.25^ab^ | 457.33 ± 5.18^a^ |
| Stage 2 | 50.75 ± 3.08^cd^ | 3388.11 ± 144.40^a^ | 3398.82 ± 142.59^ab^ | 5774.95 ± 267.43^bc^ | 424.41 ± 4.07^a^ |
| Stage 3 | 93.49 ± 21.13^a^ | 3019.82 ± 423.09^b^ | 2850.88 ± 419.24^c^ | 5487.85 ± 499.65^cd^ | 363.40 ± 14.45^b^ |
| Stage 4 | 76.07 ± 11.84^abc^ | 2850.27 ± 153.27^b^ | 2758.74 ± 198.69^c^ | 5151.80 ± 286.66^d^ | 348.66 ± 6.40^b^ |
| Stage 5 | 80.40 ± 13.77^ab^ | 3677.33 ± 248.50^a^ | 3332.91 ± 106.15^b^ | 6405.03 ± 217.06^a^ | 430.96 ± 4.37^a^ |
| Stage 6 | 54.68 ± 24.20^bcd^ | 2509.88 ± 137.78^c^ | 2238.94 ± 174.46^d^ | 4609.29 ± 630.48^e^ | 291.16 ± 6.89^c^ |
| Stage 7 | 39.11 ± 11.78^d^ | 3042.13 ± 431.46^b^ | 2630.09 ± 398.70^c^ | 5264.66 ± 409.11^d^ | 345.93 ± 14.78^b^ |

Different lowercase letters indicate values which are significantly different among the ripening stages (p < 0.05). *α*-car: *α*-carotene; *β*-car: *β*-carotene; RAE: retinol activity equivalents; dw: dry weight

Supplementary Table 2. Polar metabolite compounds from the Agung Semeru plantain at all ripening stages, resolved by a gas chromatography-mass spectrometry (GC-MS).

| **Peak** | **Retention time *(t*_R_)** | **Compound** | **Molecular formula** | **Molecular mass** | **Molecular mass - TMS** | ***m/z*** | **Ripening Stages** | | | | | | |
| --- | --- | --- | --- | --- | --- | --- | --- | --- | --- | --- | --- | --- | --- |
|  |  |  |  |  |  |  | **1** | **2** | **3** | **4** | **5** | **6** | **7** |
| 1 | 2.25 | Hydantoin | C_3_H_4_N_2_O_2_ | 100 | 100 | - | + | + | - | - | - | - | - |
| 2 | 2.43 | Oxalic acid 1 | C_2_H_2_O_2_ | 90 | 234 | 147, 73, 45, 131 | - | + | - | - | - | - | - |
| 3 | 2.51 | Oxalic acid 2 | C_2_H_2_O_2_ | 90 | 234 | 147, 73, 45, 131 | + | + | - | - | - | - | - |
| 4 | 11.53 | Phosphoric acid | H_3_PO_4_ | 98 | 314 | 73, 299, 300, 147 | + | + | - | + | + | + | + |
| 5 | 11.88 | Methylmalonic acid | C_4_H_6_O_4_ | 118 | 262 | 147, 73, 247, 215 | - | - | + | + | - | - | - |
| 6 | 13.32 | Malic acid | C_4_H_6_O_5_ | 134 | 350 | 73, 147, 233, 245 | + | + | + | + | + | + | + |
| 7 | 13.65 | Aspartic acid | C_4_H_7_NO_4_ | 133 | 349 | 282, 73, 100, 147 | + | - | - | - | - | - | - |
| 8 | 13.75 | Pyroglutamic acid | C_5_H_7_NO_3_ | 129 | 273 | 156, 73, 147, 230 | + | + | - | - | - | - | - |
| 9 | 16.51 | Ribitol / Adonitol | C_5_H_12_O_5_ | 152 | 512 | 217, 73, 147, 103 | + | + | + | + | + | + | + |
| 10 | 17.93 | Fructose 1 | C_6_H_12_O_6_ | 180 | 540 | 103, 217, 73, 307 | - | - | + | + | + | + | - |
| 11 | 18.18 | Citric acid | C_6_H_8_O_7_ | 192 | 480 | 73, 217, 201, 147 | + | + | + | + | + | + | + |
| 12 | 18.6 | Altrose | C_6_H_12_O_6_ | 180 | 540 | 217, 73, 191, 147 | - | - | + | + | + | + | + |
| 13 | 18.79 | Fructose 2 | C_6_H_12_O_6_ | 180 | 540 | 103, 217, 73, 307 | - | - | + | - | - | - | - |
| 14 | 19.15 | Fructose 3 | C_6_H_12_O_6_ | 180 | 540 | 103, 217, 73, 307 | + | + | + | + | + | + | + |
| 15 | 19.33 | Fructose 4 | C_6_H_12_O_6_ | 180 | 540 | 103, 217, 73, 307 | + | + | + | + | + | + | + |
| 16 | 19.4 | Galactitol | C_6_H_14_O_6_ | 182 | 614 | 73, 306, 217, 103 | - | - | + | + | + | + | - |
| 17 | 19.63 | Glucose 1 | C_6_H_12_O_6_ | 180 | 627 | 73, 319, 204, 147 | + | + | + | + | + | + | + |
| 18 | 19.66 | Glucose 2 | C_6_H_12_O_6_ | 180 | 627 | 73, 319, 204, 147 | - | - | + | + | + | + | + |
| 19 | 19.85 | Mannose 1 | C_6_H_12_O_6_ | 180 | 540 | 73, 204, 191, 217 | - | - | - | - | - | - | + |
| 20 | 20.01 | Glucose 3 | C_6_H_12_O_6_ | 180 | 627 | 73, 319, 204, 147 | - | - | + | + | + | + | + |
| 21 | 20.17 | Lyxose 1 | C_5_H_10_O_5_ | 150 | 438 | 73, 204, 217, 361 | - | - | - | - | - | - | + |
| 22 | 20.6 | Mannose 2 | C_6_H_12_O_6_ | 180 | 540 | 73, 204, 191, 217 | - | - | - | - | - | - | + |
| 23 | 20.68 | Lyxose 2 | C_5_H_10_O_5_ | 150 | 438 | 73, 204, 217, 361 | - | - | - | - | - | + | - |
| 24 | 21.22 | Glucose 4 | C_6_H_12_O_6_ | 180 | 627 | 73, 319, 204, 147 | + | + | + | + | + | + | + |
| 25 | 32.67 | Sucrose | C_12_H_22_O_11_ | 342 | 918 | 361, 73, 217, 437 | + | + | + | + | + | + | + |
| 26 | 33.26 | Maltose 1 | C_12_H_22_O_11_ | 342 | 918 | 204, 191, 73, 217 | - | - | + | + | + | - | - |
| 27 | 33.92 | Maltose 2 | C_12_H_22_O_11_ | 342 | 918 | 204, 191, 73, 217 | - | - | + | + | + | + | + |

TMS: trimethylsilyl; m/z: mass-to-charge ratio

Supplementary Figure 1. A typical separation profile of the targeted polar metabolite compounds from the Agung Semeru plantain in ripening stage 5, resolved by gas chromatography-mass spectrometry (GC-MS). The identification of each peak is presented in Supplementary Table 1.
